# Stigmatization toward People with Anorexia Nervosa, Bulimia Nervosa, and Binge Eating Disorder: A Scoping Review

**DOI:** 10.3390/nu13082834

**Published:** 2021-08-18

**Authors:** Lisa Brelet, Valentin Flaudias, Michel Désert, Sébastien Guillaume, Pierre-Michel Llorca, Yves Boirie

**Affiliations:** 1 Pôle R&D Santé, Jeolis Solutions, 63000 Clermont-Ferrand, France; 2 Pôle Psychiatrie B, CHU Gabriel Montpied, 63003 Clermont-Ferrand, France; vflaudias@chu-clermontferrand.fr; 3 EA 780 NPsy-Sydo, Université Clermont Auvergne, 63001 Clermont-Ferrand, France; 4 Laboratoire de Psychologie Sociale et Cognitive (LAPSCO), Centre National de la Recherche Scientifique (CNRS), Université Clermont Auvergne, 63003 Clermont-Ferrand, France; michel.desert@uca.fr; 5 Département des Urgences Psychiatriques, Hôpital Lapeyronie, CHRU Montpellier, 34295 Montpellier, France; s-guillaume@chu-montpellier.fr; 6 Institut de Génomique Fonctionnelle (INSERM), Centre National de la Recherche Scientifique (CNRS), Université de Montpellier, 34094 Montpellier, France; 7 CMP-B CHU, Clermont Auvergne INP, Institut Pascal, Centre National de la Recherche Scientifique (CNRS), Université Clermont Auvergne, 63000 Clermont-Ferrand, France; pmllorca@chu-clermontferrand.fr; 8 Centre Troubles des Conduites Alimentaires (TCA), Service de Nutrition Clinique, Unité de Nutrition Humaine, CHU Gabriel Montpied, CRNH, Université Clermont Auvergne, 63003 Clermont-Ferrand, France; yboirie@chu-clermontferrand.fr

**Keywords:** eating disorders, anorexia nervosa, bulimia nervosa, binge eating disorder, stigmatization, treatment compliance

## Abstract

Research about stigmatization in eating disorders (EDs) has highlighted stereotypes, prejudices, and discrimination against people with EDs, as well as their harmful effects on them, including self-stigma and a difficult recovery process. Whereas a recent review focused on the consequences of ED stigma, our work aimed to provide a broader synthesis of ED stigma, including its consequences, but also its content and distribution. More precisely, we focused on three EDs—namely, anorexia nervosa, bulimia nervosa, and binge eating disorder. Based on a systematic search of four major databases in psychology, the present scoping review includes 46 studies published between 2004 and 2021. We did not conduct any quality assessment of the studies included, because our aim was to provide a wide-ranging overview of these topics instead of an appraisal of evidence answering a precise research question. The review confirmed the existence of a common ED stigma: all individuals affected by EDs reviewed here were perceived as responsible for their situation, and elicited negative emotions and social distance. However, our review also depicted a specific stigma content associated with each ED. In addition, the demographic characteristics of the stigmatizing individuals had a notable influence on the extent of ED stigma: men, young adults, and low-income individuals appeared to be the most stigmatizing toward individuals with EDs. It is important to note that ED stigma had a negative effect on individuals’ eating disorders, psychological wellbeing, and treatment-seeking behavior. There is an urgent need for further research on the adverse effects of ED stigma and its prevention.

## 1. Introduction

According to the American Psychiatric Association (https://www.psychiatry.org/patients-families/eating-disorders/what-are-eating-disorders, accessed the 29 July 2021), eating disorders (EDs) are behavioral conditions characterized by severe and persistent disturbance in eating behaviors, affecting physical, psychological, and social functions. In addition to physical and medical health considerations, researchers have recently focused on stigmatization against individuals affected by an ED [1,2,3], because of its multiple negative consequences, (e.g., increased ED symptoms, decreased treatment-seeking behaviors, etc.) [4,5,6]. More precisely, those works have also investigated the content and the distribution of ED stigma [7,8].

### 1.1. Stigma and Mental Disorders

Stigmatization of social groups is common worldwide [9,10]. Stigmatized individuals are treated differently, excluded, or even socially rejected because they do not meet specific societal standards (e.g., skin color, physical disability, or age) [11]. Since EDs are mental disorders, one could argue that the stigma of EDs could be addressed through the mental illness stigma [12,13]. The scientific literature on mental disorders suggests that stigma associated with mental illness is prevalent [12,13].

Social psychologists distinguish between three stigma components: a cognitive, an emotional, and a behavioral component. To be more precise, stereotypes constitute the cognitive component of stigma. They are either positive or negative beliefs shared by individuals about social group members (e.g., believing that all online gamers are socially incompetent, lazy, and unattractive) [14]. Prejudice is the emotional component of stigma, comprising unfavorable attitudes (e.g., anger, fear, disgust, pity) toward someone because of their social group membership [15]. Finally, discrimination refers to the behavioral component of stigma. Discrimination is traditionally defined as negative behaviors directed toward individuals because they belong to a specific social group. Such actions serve to disadvantage people in many contexts [16,17]. The general term stigma typically indicates that at least one of these components is present.

Regarding mental illnesses, studies have highlighted the presence of stereotypes, prejudices, and discrimination against people with these disorders. For example, individuals with mental disorders are stereotyped as dangerous, childish, incompetent, weak, and responsible for their condition [18]. Concerning prejudice, Angermeyer and Dietrich [12] observed prosocial reactions toward people with mental disorders (e.g., feeling sorry for them, expressing the desire to help), but also discomfort, feelings of uncertainty, and fear reactions. In the same way, Corrigan and Watson [19] found that individuals with mental disorders were less appreciated than individuals with physical illnesses. Hipes et al. [20] highlighted discriminatory behaviors showing that fictitious job applicants described as fully recovered from a mental health condition received fewer call-backs than fictitious candidates who had recovered from a physical injury.

As shown above, people with mental illness—and, thus, people with EDs—can be stigmatized by others, with detrimental consequences to them [18]. Additionally, the experience of stigma can lead people with mental disorders to self-stigmatize; many consider themselves weak and incompetent, experience low self-efficacy and low self-esteem, and do not pursue work or other independent-living goals [19]. Moreover, those who had the most stigmatizing experiences were also the most reluctant to seek professional support, and had the poorest treatment adherence. This reluctance to seek help appeared to be more prominent among men, seniors, African Americans, and Hispanics than women, young people, and Caucasians experiencing mental disorders [18].

While these previous studies highlight a general stigma against all mental illnesses, there are also differences and specificities depending on the mental illness involved. For instance, unpredictability and dangerousness were traits explicitly associated with individuals affected by schizophrenia or alcoholism [12,13]. Avoiding personal contact with individuals with a mental disorder was also more often observed in schizophrenia, alcoholism, or drug addiction, compared to depression or anxiety disorders [12]. In addition, there is interindividual variability in the prevalence of mental illness stigma and, more specifically, in the occurrence of prejudice. For example, Angermeyer and Dietrich [12] found that age, education level, and familiarity with mental disorders played a role in the extent of mental illness stigma. Therefore, although EDs are mental illnesses, it seems important to specifically address ED stigma—namely, its content, distribution (i.e., prevalence in different social groups), and consequences.

### 1.2. Stigma and Eating Disorders

Eating disorders are recognized as stigmatized mental disorders [12,13]. The Diagnostic and Statistical Manual of Mental Disorders (5th ed.; *DSM-5*) [21] defines three specific EDs—namely, anorexia nervosa (AN), bulimia nervosa (BN), and binge eating disorder (BED). Two additional categories of ED are also included in the *DSM-5*: other specified feeding and eating disorders (OSFED, e.g., atypical anorexia; night eating syndrome, etc.), and avoidant restrictive food intake disorder (ARFID). However, we have chosen to focus on AN, BN, and BED as the main EDs investigated in the literature [4,22,23,24].

Regarding ED stigma, Crisp et al. [13] examined perceptions of various mental disorders, including EDs (with no distinction between AN, BN, and BED). They observed that more than a third of respondents blamed people with EDs for their situation, thought that people with EDs would be able to pull themselves together if they wanted to, and found communication with them challenging. Nevertheless, the study demonstrated that stigmatizing attitudes toward people with EDs were less common than toward people with schizophrenia, alcoholism, and drug addiction. Negative beliefs shared by the general public engendered negative emotions (anger and fear) toward people with EDs and avoidant behaviors (e.g., withholding assistance with work or housing opportunities) [18]. Since then, some experimental studies have specifically investigated the content of ED stigma, showing the existence of negative beliefs, e.g., responsible for their condition [25,26], negative attitudes [27,28,29], and social distance toward people with EDs [25,30,31,32].

Other work has focused on the distribution of ED stigma. It was found that men are the most stigmatizing toward people with EDs [26,33]. Importantly, Thompson-Brenner et al. [34] found that clinicians themselves also expressed negative beliefs (e.g., holding people with EDs responsible for their condition) and negative emotions (e.g., frustration, anger) toward people with EDs. The extent of ED stigma in clinical staff was also linked to individual characteristics, such as their clinical discipline, professional experience with EDs, and gender. More precisely, inexperienced clinicians, nurses compared to physicians, and men compared to women were more likely to show adverse and stigmatizing reactions to people with EDs.

Concerning the consequences of ED stigma, Foran et al. [4] conducted a recent systematic review of nine studies and found that ED stigma predicts negative outcomes for people with EDs on multiple levels: psychological, social, physical, and health behaviors. Indeed, the review indicated that ED stigma can lead to depressive and self-esteem symptoms, social alienation and social withdrawal, poor physical health, and greater ED symptoms, but also greater avoidance of treatment-seeking behaviors, increasing the physical (e.g., cardiac issues), psychological (e.g., distress), and social (e.g., poorer quality of life) complications associated with EDs [35,36] Consequently, by highlighting these negative consequences of ED stigma, this review and related works shed light on the importance of studying ED stigma to better understand and act on it.

Finally, one could expect that individuals with EDs would also be affected by weight-related stigma, which refers to any situation where a person feels treated differently, rejected, or excluded because their weight differs from cultural norms [37,38,39,40]. Indeed, people with a visible stigma (e.g., obesity) are more likely to be discredited than those with a non-visible stigma [11]. However, EDs do not necessarily involve physical changes; thus, ED stigma and weight stigma are only partially correlated [22,41]. Consequently, the present scoping review focuses specifically on ED stigma, but not weight stigma.

### 1.3. Aims

Empirical studies and reviews have demonstrated variation in the content and consequences of stigma associated with different mental disorders. One criticism is that most of this work has concentrated on psychotic or mood disorders such as schizophrenia or depression, while EDs have been relatively overlooked. The scarce research that has recently focused on ED stigma typically either examined just one ED (e.g., AN) [27,28]; did not necessarily distinguish between AN, BN, and BED; or addressed only a single aspect of ED stigma: either its content, its stigmatizing sources, or its consequences [4,7,42]. For example, the recent systematic review conducted by Foran et al. [4] only investigated the consequences of ED stigma.

Focusing on the consequences of ED stigma is crucial because of its severe impact on people with EDs (e.g., depressive symptoms, increased ED symptoms, decreased treatment-seeking behaviors). Nevertheless, we thought it was important to go further in describing the content and the distribution of this stigma, as this may be useful in understanding these consequences and identifying ways to reduce them. We therefore conducted a scoping review [43] of all recent articles to provide a broader overview of ED stigma, addressing all of its dimensions—namely, its content, distribution, and consequences.

We believe that a new review on this topic will be helpful to increase healthcare professionals’ awareness and understanding of EDs in order to improve their treatments [36]. An overview of the recent literature about ED stigma can also help to identify research gaps and ways to address ED stigma.

Thus, the purpose of this scoping review was to (a) synthesize current knowledge on *common* and *specific* stigma content of AN, BN, and BED in the form of stereotypes, prejudice, and discriminatory behaviors against people with EDs; (b) identify the sociodemographic characteristics (i.e., age, gender, education, income, etc.) associated with increased stigmatization against people with EDs; and (c) summarize the impact of ED stigma on individuals with EDs in terms of self-stigma, eating disorders, psychological disorders, treatment-seeking behavior, and therapeutic compliance.

## 2. Methods

In accordance with the Preferred Reporting Items for Systematic Review and Meta-Analysis extension for Scoping Review (PRISMA-ScR) 2018 statement [43], this scoping review systematically searched for all recent studies investigating ED stigma toward people with AN, BN, or BED, including its content, distribution, and consequences.

### 2.1. Eligibility Criteria

Inclusion criteria: All experimental studies included were written in English and published in peer-reviewed scientific journals. We included only studies investigating AN, BN, or BED. Because ED stigma is a novel area that has only begun to be explored [4,22], we searched for studies published from 2000 onwards.

Regarding the content of ED stigma, we included studies that looked at the stereotyping of, prejudice against, and discriminatory behaviors toward people with AN, BN, and BED. With regard to the distribution of ED stigma, we included studies that examined the degree of stigma toward people with EDs according to participants’ social category (e.g., age, gender, income, education level, etc.). Concerning the consequences of ED stigma, we included studies that examined the physical, psychological, medical, and social impacts that ED stigma can have on people with EDs. Participants in these studies could be of any age, gender, or nationality, and could come from the general population, health professionals, or ED patients themselves. Moreover, we made no restrictions on the method used to assess ED stigma (e.g., self-administered questionnaires, structured interviews, vignette paradigm).

Exclusion criteria: We excluded literature reviews, commentaries, papers describing psychometric scale validation, academic theses, books, articles with no English language version, and any work published before 2000. Moreover, this review does not include phenomena related to but different from ED stigma, such as mental illness stigma and weight stigma. Finally, we focused only on studies dealing with stigma toward people with EDs and its impact on them. Stigma can also exist toward the family and relatives of people with mental illness [44,45], but this review did not address this topic.

### 2.2. Information Sources, Search, and Study Selection

A computerized search of the PsycINFO, PubMed, ScienceDirect, and Google Scholar databases was conducted to identify studies published on ED stigma. We successively searched in Title the different combinations of the following terms: “eating disorder” OR “disordered eating” OR “anorexia” OR “bulimia” OR “BED” OR “binge eating disorder”, AND “stigma” OR “stigmatization” OR “stereotype” OR “opinion” OR “prejudice” OR “attitude” OR “discrimination” (for example, in PsycINFO one combination was: anorexia nervosa (Title) “AND” Stereotype (Title)). No term was excluded (i.e., no use of the “NOT” option). No reference lists were consulted. Thus, no additional studies to the selected paper were added.

These data sources, searches, and study selection were carried out by one researcher (L.B.) in November 2019, with an update in May 2021.

### 2.3. Data Extraction

At this step, the total of included studies (*n* = 46) was classified according to the type of ED concerned—AN, BN, and/or BED—and the dimension of stigma studied: content, distribution, and/or consequences. Information on study location, participants, and ED stigma measures used were extracted (see Table 1 and Appendix A). The main results of the 46 studies included in this scoping review are reported in a narrative manner. For the content of ED stigma, the data of articles included were grouped into three sections—stereotypes, prejudice, and discrimination—that concern, respectively, the beliefs, the emotions/affect, and the behaviors toward people with EDs. Regarding the distribution of ED stigma, the results were reported in 2 sections: the social categories that are most stigmatizing toward people with EDs, and those that are least stigmatizing. We categorized people/groups as “the most” and “the least” stigmatizing according to the authors of studies included in our review. These categories include gender, age, socioeconomic status (e.g., education and income levels), weight, and ethnicity, but also people who are familiar/unfamiliar with EDs, people who are informed/uninformed about EDs, and people who believe/do not believe in a “just world”. Individuals familiar with EDs are defined as people who have experienced or are currently experiencing an ED, or they may have an acquaintance (e.g., relative, friend, patient) with an ED or a history of ED [26,46]. People who are informed about EDs are individuals who have knowledge about the disease, its symptoms, and/or its treatments without necessarily being familiar with it [47]. People who believe in a just world represent individuals who think that we get what we deserve, and we deserve what we get [48]. Studying the disparities in ED stigma among these groups would be helpful to determine which populations require anti-stigma intervention, and to guide the content of these programs (e.g., increasing knowledge, decreasing belief in a just world, etc.). Finally, concerning the consequences of ED stigma, articles that examined similar outcomes related to ED stigma experiences and recovery were grouped into 3 sections: the patients’ stigma perception, self-stigma, and the impact on ED symptoms and severity.

### 2.4. Risk of Bias

As mentioned earlier, our study selection was not strictly limited to a specific assessment method of ED stigma. Moreover, we did not conduct a quality assessment to critically appraise the quality of evidence provided by the primary studies. Therefore, the studies included in our review might suffer from some heterogeneity regarding their methodological design and their respective quality.

## 3. Results

### 3.1. Characteristics of Included Studies

**Figure 1:**
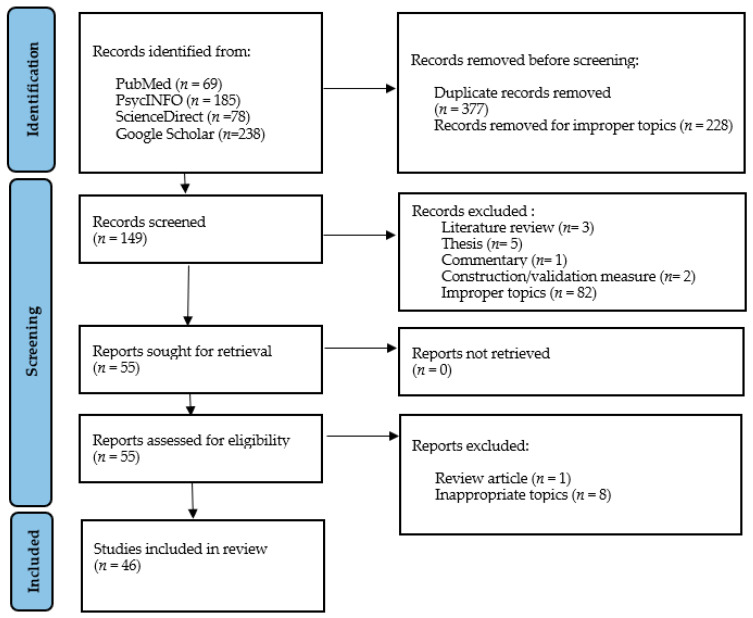
Flowchart of the inclusion procedure for the review.

In total, the search produced 570 articles published between 2000 and 2021. Of these, 143 occurred in more than one database; thus, 377 studies remained after duplicates were manually removed. Of the remaining articles, 228 were removed for improper topics (mainly assessment of participants’ attitudes to eating disorders and assessment of eating disorders in response to weight stigma). After screening the remaining 149 abstracts, a further 94 articles were excluded based on the inclusion/exclusion criteria mentioned above. Most were excluded for improper topics, e.g., studies on weight stigma, the relationship between EDs and body shape concerns, the effectiveness of anti-stigma interventions, ED treatments, etc. None were removed due to low quality of outcomes, low quality of study design, or wrong population of interest. Following full-text examination, 46 relevant studies were selected for this review (see Figure 1 for a PRISMA diagram summarizing our inclusion procedure) [76].

The included studies were published between 2004 and 2021, and just over half (27/46) had been published since 2015s (see Table 1 for the main information about the included studies, and see Appendix A for more details on the tools used). Twenty-nine studies examined the *content* of ED stigma. Some studies reported stigma content for a specific ED: eight focused on AN, six on BN, and one on BED. Other studies compared stigma content between different EDs: five contrasted AN and BN, one AN and BED, one BN and BED, and seven analyzed differences between all three EDs. Twenty-three studies examined the *distribution* of ED stigma. Most studies focused specifically on AN (*n* = 6), on BN (*n* = 4), on both AN and BN (*n* = 7), or on all three EDs (*n* = 4). Only one study examined AN and BED, and one for BN and BED. None of the studies focused uniquely on BED. Ten studies investigated the *consequences* of ED stigma; half of these discussed repercussions for all three disorders. The remaining studies focused specifically on AN (*n* = 3), BN (*n* = 1), or AN and BN combined (*n* = 1).

Most studies explored stigma among the general population, patients with EDs, and healthcare professionals. It is worth noting that sampling bias is likely, as many studies (20/46) included college students—particularly psychology students (*n* = 12), where women are overrepresented compared to men.

The selected studies used the vignette paradigm (*n* = 26), questionnaires (*n* = 39), and semi-structured interviews (*n* = 6) to investigate ED stigma. In the vignette paradigm, participants read one or more fictional stories about targets with a specific ED and answer open-ended questions or complete validated standardized questionnaires about the target. These questions assess recognition, perception, and knowledge of EDs, and beliefs, attitudes, and behaviors toward persons with EDs to examine stereotyping, prejudice, and discrimination toward this population—for a vignette paradigm example, see [1].

### 3.2. Content of Stigma

As stereotypes, prejudice, and discrimination are established factors contributing to stigma, we explored these three dimensions to characterize ED stigma. The following section provides a synthesis of those findings (see Appendix B for an overview of stigma content for each ED).

#### 3.2.1. Stereotypes

The selected studies focused on five aspects of ED stereotyping: the notion of responsibility, character traits, gender attribution, disease severity and control, and supposed causes.

Responsibility: The general population and most healthcare professionals perceived people with EDs as blameful and responsible for their situation [25,26,28,29,31], for studies on healthcare professionals see [46,52,63,66]. This trend was especially notable for BN compared to AN [66], and for BED compared to AN [33] and AN/BN [2,63]. Individuals with EDs were more likely to be associated with personal responsibility than those with depression or a major depressive episode (MDE), but less so than those with obesity [1,2,28,32,52]. Indeed, an obese individual without BED was more likely to be blamed for his or her situation than an obese individual with BED [51]. In addition, one recent study found that a target with no mental disorder or weight-related problems was blamed more for their situation (i.e., eating more fast food during stressful times and spending money on these foods) than a target with AN, BN, or BED [2]. Patients’ age seemed to moderate this responsibility belief. For instance, participants rated a fictitious 12-year-old boy less responsible for the onset of his BN than the same boy aged 24 [50]. Thörel et al. [2] found no significant effect of target ages on stigmatizing attitudes, but the ages used were older: 19 and 39 years, respectively.

Character traits: Studies in the general public found that participants attributed negative traits to people with EDs, but less so for those with BED [1,54]. However, targets with BED have been described with less desirable personality traits (e.g., weak, lazy, and careless) than targets without BED [51]. The general population associated people with BED with a larger body [51], and rated people with AN as dangerous, incompetent, able to pull themselves together if they chose to, hard to talk to [25,29,52,58], and considerably attention-seeking [28,29,52,59]. The perception of people with EDs as self-destructive was also commonly held, especially for those with BN [58,66]. Healthcare professionals demonstrated similarly negative beliefs about people with EDs—e.g., that they are manipulative, disrespectful, deceitful, and non-compliant with treatment (for a study on nurses, see [55])—and people with AN, i.e., unreliable (for a study on nursing students, see [26]).

Finally, some studies noted that targets with other mental or physical disorders elicited a more favorable personality trait assessment than targets with EDs [56], AN, [27,29,32,59] or BED [1]. However, this trend does not appear to be uniformly held across all personality traits. Some studies found that targets with AN were rated more negatively than those with other mental disorders on some dimensions (i.e., intelligence and communication), but more positively on others (e.g., dangerousness, motivation, enthusiasm; for a comparison with schizophrenia, see [29]; for a comparaison with depression, see [27,59]).

Gender attribution: AN was perceived to primarily affect women [3,63], as was BN [66], but for non-significant results see [34,51]. For BED, the reverse trend was observed, although the association with men was not statistically significant [3,63]. In a recent study on Turkish school counselors, all three EDs were more associated with women than men compared to other disorders. Female counselors made this assumption more than male counselors [56].

Disorder severity and control: The general public recognized BN as a severe and disabling condition that is difficult to treat [31,60], and perceived it as more serious than BED [62]. People rarely minimized BN or perceived it as beneficial [25,26]. In general terms, this was also true in AN [25]; however, some participants still praised people with AN for their ability to control and lose weight [30]. Overall, the general public appeared optimistic about people’s ability to control and recover from EDs. Compared to individuals with other mental or physical disorders, AN and BED were perceived more as psychopathological [33], and EDs as a long-term illness where individuals affected by these disorders have less control [56]. In contrast, some studies have shown that those with BED were thought to have more personal control over their condition [63]. People with AN were perceived as more capable of composing themselves and recovering [36], but for contradictory results in athletes, see [47].

Supposed causes: EDs’ causes are mainly internalized (i.e., perceived to be the result of personal responsibility). Lack of self-discipline was noted as a common cause for all three disorders [1,29,57,68], although this was less pronounced for AN [1,2,66], and more for BED [1,2]. BED was linked to a lack of self-control and willpower [1,62]. In some studies, participants attributed distinct and specific causes to AN and BN. Vanity and the desire for attention were noted as the leading causes of AN [28,29,30,52]. Conversely, BN was linked with low self-esteem [61].

When external causes (i.e., context-related influences) were noted, participants mainly mentioned a lack of social support [29,57,68]. Media was also reported as a main causal factor in AN and BN, while genetic factors were not perceived as being involved [26]. Family problems were also mentioned for EDs [56]. Specifically, parents’ role was also considered in AN and BN [29,57,68], but not for BED [68]. The general public also associated AN and BN with specific external causes: AN with social influences [29], and BN with sexual abuse and being overweight or obese during childhood or adolescence [61].

Compared to other mental or physical disorders, AN was more likely to be linked with low self-discipline [29,32,57], lack of social support, and poor parental support [29,32]. AN was rarely attributed to genetic or biological factors [29,32,57]. For BN, the desire for attention was more widely reported [56].

#### 3.2.2. Prejudice

The studies we reviewed indicated that the general public feels conflicting emotions about people with AN. Study participants expressed negative emotions such as irritation and anger, lacked sympathy, and felt uncomfortable in personal interactions with people with AN [28,29,30,32]. On the other hand, people with AN aroused admiration and inspired a degree of willingness to imitate them [28], but for contradictory results see [27]. Imitation was also reported for BN targets [52], although BN was not perceived as admirably as AN [31,60]. People with BN seemed to inspire sympathy [60] and more friendly attitudes [52]. People with BED received equal or more negative attitudes, e.g., compared to those with AN and BN [54,63]; and people without BED [51]. However, AN and BN elicited more distrust than BED [2,33]. AN was also subject to more distrust than other eating problems (i.e., restrictive eating, emotional eating, picky eating, and ARFID) [33].

Studies on healthcare workers indicated that professionals might share the public’s negative emotions about EDs. They reported a loss of motivation to deliver or manage care to these patients and expressed negative emotions such as discomfort, frustration, fear, stress, anger, exasperation, and displeasure [46,53,55].

Overall, compared to other mental or physical disorders, the reviewed research demonstrates that EDs provoke fewer positive reactions in both the general population [2,28,57] and healthcare professionals [49]. Nevertheless, individuals affected by EDs elicited less distrust than those with an MDE [2]. In addition, people with AN inspired more admiration, and people with BN more friendly attitudes, than those with depression [28,52], and study participants reported feeling more comfortable interacting with people with AN than those with schizophrenia [29].

#### 3.2.3. Discrimination

At the behavioral level, there is some evidence that people maintain social distance between themselves and people with EDs. Study participants reportedly avoided personal contact with people with AN [25], and were reluctant to interview them for a job [30]. A desire for social distancing was also expressed toward people with BN [31,32]. For BED, participants’ desire for social distance from targets with BED was low, and not significantly different when compared to targets without BED [51]. Study participants were more likely to want to interact with people with BED than those with AN or BN [63], and expressed less desire for social distance from them [2], but for no significant difference in social distance assessment for all three ED populations, see [54].

The public’s tendency to socially distance seems to be a common finding for a range of mental disorders, e.g., AN, schizophrenia, depression, see [2,57]. One study reported that people with AN produced less social distance than people with depression in the general public [28], while the opposite was found among healthcare professionals [3]. Compared to physical disorders, the general population reported more social distance from targets with AN [32] and all three EDs [2]. Similarly, McNicholas et al. [3] found that healthcare professionals preferred to manage people with type 1 diabetes rather than those with AN. Finally, Lupo et al. [26] showed that nursing students reported a high level of social distance from people with AN, while that for people with BN was low.

### 3.3. Distribution of Stigma

Some studies included in the review found that ED stigma is more likely to be expressed in some populations than others (see Table 2 for details).

#### 3.3.1. Most Stigmatizing Individuals

Men held stereotypes and prejudice against people with EDs to a greater extent than women [26,33]. Men were more likely than women to have stereotypes such as believing in patients’ responsibility [7,31,33,47] and attributing the condition to internal causes, e.g., narcissism, lack of willpower, attention-seeking, [7,58,59,66,67]; for one study on BED see [62]. They were also more likely to minimize the challenge and severity of AN and BN [27,31,33,47,58,66]. Men also expressed more prejudice than women, exhibiting less prosocial and friendly attitudes toward people with EDs [65,66,67]. However, the findings on discriminatory behaviors are inconclusive. Some studies found no gender difference in ED stigma [25,31], but Makowski et al. [67] found that women reported more social distance from people with EDs. A recent study found that AN stigma was not predicted by gender *per se*, but rather by compliance with masculine gender norms, which resulted in more stigmatizing behavior toward people with AN [8].

A few studies examined the influence of age, educational background, and income on ED stigma. Overall, young adults were more likely than older adults to express stigma or seek social distance from people with AN or BN [25,31,61,64]. The same pattern of results was observed for individuals with low income compared to those with high income [31,64]. Results were mixed for educational background. Two studies found that individuals with a low education level were more stigmatizing than those with a high education level [31,64], but in two others, the effect of education on stigma was not statistically significant [60,61].

Finally, one study observed that individuals who strongly believed in a “just world” were more stigmatizing toward people with EDs than those who did not share this belief [68].

#### 3.3.2. Least Stigmatizing Individuals

Several studies have examined the impact of familiarity with EDs on stigma. The studies included in this review indicated that individuals familiar with EDs held few stigmatizing attitudes toward people with EDs (for AN and BN, see [25,26,46]). Research also suggested that familiarity was associated with less discomfort and greater sympathy toward patients with EDs [27,32,60], but for a non-significant finding see [46]. At the behavioral level, Caslini et al. [25] observed that people with EDs themselves were less likely to desire social distance from people with BN. Nevertheless, one study indicated that those familiar with EDs were more likely than those unfamiliar to think that BN is caused by low self-esteem and sexual abuse. Individuals familiar with EDs were also more likely to minimize BN and identify benefits associated with the condition [61]. Only one study found no impact of familiarity on the extent of stigmatizing attitudes against people with BN [47]. Caslini et al. [25] offer one potential explanation for this disparity in the literature, noting that the influence of familiarity might depend on the proximity to the individual with an ED. Stigma was less prevalent if the person with AN or BN was a close relative (e.g., boyfriend or girlfriend vs. cousin).

A small number of studies focused on people with significant ED symptoms (but without a formal diagnosis) or on people who were knowledgeable about EDs. These two populations attributed fewer negative stereotypes—such as selfishness, lack of self-control, and responsibility—to people with EDs compared to people with few ED symptoms or limited knowledge about EDs [30,47,66]. At the emotional and behavioral levels, people with significant ED symptoms also reported less irritation and less desire for social distance than people with few ED symptoms, demonstrating a stronger inclination to befriend people with AN [30].

Finally, a handful of studies focused on factors like participants’ degree program, ethnic group, and weight category. Psychology students, Caucasian participants, and underweight individuals had less stigmatizing attitudes toward people with EDs than students in other degree programs [7,25], African-American participants [66], and normal-weight or overweight individuals [25].

### 3.4. Consequences of Stigma

**Figure 2:**
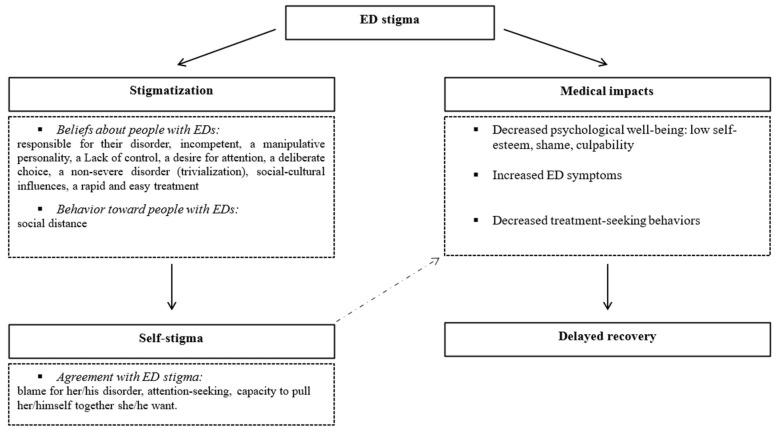
Consequences of stigma against people with eating disorders (EDs).

Compared to studies on ED stigma content and distribution, very few studies have examined the consequences of ED stigma (*n* = 10). The reviewed research suggests that people with EDs often experience ED stigma, and that stigmatization can have significant impacts (see Figure 2).

#### 3.4.1. Stigma Perception

Few studies have examined ED stigma from the patient’s perspective. The studies included in this review mainly focus on female patients with AN. Most women with AN reported ED stigmatization by the general public [6]. People with AN felt that they were stereotyped in public opinion, and that others blamed them for their situation and attributed negative characteristics to them, such as incompetence, lack of self-control, and manipulative personality traits [6,69,73]. People with AN also found that others proposed superficial causes to explain AN [69], and trivialized their disorder, as well as their treatment and their recovery [69,73]. People with AN noted discriminatory behavior, finding that people distanced themselves from them [6,69]. Finally, Bannatyne and Stapleton [71] highlighted that people with AN also experienced negative beliefs (e.g., blame, trivialization) and negative reactions (e.g., frustration, no empathy) from healthcare professionals.

#### 3.4.2. Self-Stigma

ED stigma seems to lead to self-stigma among people with EDs. Maier et al. [6] found high rates of internalization of others’ negative beliefs (e.g., blame, attention-seeking, the ability to pull themselves together if they wanted) in women with AN. Self-stigma was found to be an independent predictor of having an undiagnosed ED [74], and has been linked to reduced treatment-seeking and a more negative attitude toward recovery [72]. Among patients with AN, Dimitropoulos et al. [42] found that high self-stigma and perceived stigma toward family members were negatively correlated with recovery attitudes. Griffiths et al. [73] reported that greater resistance to stigma (i.e., being able to counteract stigma or remain unaffected) was associated with less severe depressive symptoms, enhanced self-esteem, and a more positive attitude toward treatment-seeking.

#### 3.4.3. Impact on ED Symptoms and Severity

ED stigma can have harmful psychological consequences, such as low self-esteem and feelings of shame and culpability (for a study on BN, see [70]). In addition, stigma can affect the course of the ED itself. Griffiths et al. [5] found that more frequent experience of ED stigma was associated with higher levels of ED psychopathology. A further study examining the underlying mechanism for this association demonstrated that ED stigma led to greater alienation and social withdrawal which, in turn, predicted greater symptom severity [75]. A third potential adverse effect of stigma is its impact on the treatment of EDs. Griffiths et al. [5] observed that the experience of stigma was associated with longer illness duration and reduced treatment-seeking. Maier et al. [6] noted that a substantial proportion of people with AN waited a long time before visiting a physician and starting treatment because they were afraid of being stigmatized. The average period between disease onset and the first medical consultation or treatment initiation was around 8–9 months.

## 4. Discussion

The primary aim was to review and synthesize the evidence on ED stigma, producing a comprehensive update on stereotypes, prejudice, and discriminatory behaviors toward people with EDs. We examined ED stigma in a broad sense and explored stigma content associated with specific EDs (i.e., AN, BN, and BED). The second objective was to describe the distribution of ED stigma—specifically, to understand which groups were more likely to stigmatize people with EDs and which groups were more tolerant. The final objective was to summarize the known consequences of ED stigma—specifically, the impact on the healthcare of people with EDs.

### 4.1. Present Contributions and Future Research

Regarding the *content* of ED stigma, there is strong evidence of stigma against patients with AN, BN, and BED. Overall, people with EDs were perceived by the public as being responsible for their disorder. Causal attributions included a lack of self-discipline (dispositional cause) and a lack of social support (situational cause). Examination of the affective and behavioral dimensions revealed that negative attitudes and social distance toward patients with EDs are also common.

These data are consistent with findings on mental illness stigma in general. Indeed, previous studies have shown that the general public believe people with mental illnesses are responsible for their condition [13,18], display adverse reactions like discomfort or fear [12,77], and avoid personal contact with them [12,20].

This review has also highlighted that some beliefs and attitudes were more strongly associated with specific EDs. First, AN was generally perceived as a way of garnering attention from others and considered easy to overcome. Only people with AN provoked both irritation and a desire to imitate. Secondly, self-destruction, low self-esteem, sexual abuse experience, and being overweight or obese in childhood or adolescence were uniquely associated with individuals affected by BN. This disorder was rated severe and generated more sympathy. Thirdly, people with BED were less likely to elicit negative character assessments than those with AN or BN, but were nevertheless associated with equally or more negative attitudes, except for distrust. People with BED were more likely to be held responsible for their condition. Lack of self-control as well as lack of willpower were also strongly associated with BED.

These data support previous findings of variation in stigmatizing content between different mental health conditions. For example, unpredictability and dangerousness were uniquely associated with people with schizophrenia compared to people with other mental illnesses [12,13,78]. However, above all—as mentioned in the introductory section—the difference in stigma content between AN, BN, and BED could be explainable by their differences in stigma visibility and conformity to cultural norms [11]. One could hypothesize that people with BN may have engendered more sympathy because BN is sometimes less visible than AN and BED. People with AN may have triggered more admiration because their body shape could be more consistent with the predominant ideal of thinness [79,80]. Conversely, people with BED may have been the target of blame attribution because their body shape might differ from cultural weight standards.

Accordingly, BED stigma content showed overlap with weight stigma toward overweight people [15,81,82], which has negative psychological and behavioral consequences. Indeed, overweight people are victims of stereotypes. Horsburgh-McLeod et al. [83], for example, found that participants’ descriptions were more negative for an overweight target than for the same target with a normal weight. Obese people were also less valued. Using photographs of children, Musher-Eizenman et al. [84] found that child participants were less likely to choose photographs of overweight children as potential friends than photographs of thin or average weight children. Furthermore, this population is also disadvantaged in important areas of life. A field experiment demonstrated that hiring managers were less likely to invite obese individuals than normal-weight individuals to a job interview, even when applicants’ cover letters and curriculum vitae contained similar skills and qualifications [85]. Moreover, studies have shown that weight stigma has harmful consequences, e.g., depression, eating symptoms, [86,87,88]. However, a recent study found no evidence for additive effect of BED stigma and weight stigma [51]. The presence of BED limited and moderated weight stigma. Indeed, authors observed that an obese target with BED elicited fewer stereotyping characteristics (i.e., negative personality traits and blame) than an obese target without BED. Conversely, a study has shown that overweight people with BN received more blame and more weight problem attributions (compared to severe psychiatric disorder attributions) than underweight people with BN [89].

Our finding of public stigma around the causation of EDs is consistent with previous research demonstrating correlations between sociocultural factors and EDs. For example, one recent study found that peer pressure was a significant predictor of the risk of EDs [90]. Therefore, social causes are perhaps more likely to be associated with AN, because the body shape of people with AN might be more congruent with sociocultural weight and beauty standards.

Study participants’ gender attributions were consistent with ED prevalence statistics, which show a higher prevalence for women than for men, although this is less pronounced for BED. In 2017, the National Institute of Mental Health reported a male/female ratio of 1:3 for AN, 1:5 for BN, and 1:2 for BED [91]. However, those statistics should be treated with caution. Indeed, several studies have noted that men with EDs were underdiagnosed because they avoided care services out of shame of being affected by a “female disorder” [5,92], leading to the overrepresentation of females in ED prevalence. Redefinition of the ED diagnostic criteria in the *DSM-5*, which now capture more male eating problems (e.g., removal of the amenorrhea criterion), might resolve this issue to some extent.

Our review has also highlighted that healthcare professionals were not immune to ED stigma and commonly endorsed public stigma, which is consistent with previous research indicating that healthcare practitioners were a source of mental illness stigma [78,93] and weight stigma [94,95]. The discomfort and reluctance of healthcare professionals to manage patients with EDs—especially patients with AN—is mainly explained by practitioners as a lack of knowledge, experiences, and equipment to treat them [3,46]. People with EDs have a higher risk of mortality than their counterparts in the general population [96], especially individuals with AN [97]. The latter also have the highest mortality risk compared to people with other mental disorders, such as schizophrenia, depressive episode, dementia, etc. [98]. This high risk of mortality in EDs may create prejudice among providers, but study of these links should be pursued.

Regarding the *distribution* of ED stigma, the past 15 years of research have principally shown that personal characteristics play a role in the extent of ED stigma. Most studies indicated that men tend to be more stigmatizing against people with EDs than women. However, this finding was qualified by a recent study that showed that stigmatization might be linked to compliance with masculine gender norms, and not gender *per se*. Several studies also noted an effect of age and income level on the extent of ED stigma. Young adults and low-income individuals were found to be more stigmatizing than older adults and those with higher incomes. The evidence for a link with educational attainment is mixed. Whereas some studies observed more stigmatization from individuals with less education, others found no significant difference based on education level. The effect of ED familiarity was less ambiguous. People who were familiar with EDs or knowledgeable on the topic and those who had experienced significant ED symptoms had fewer negative attitudes toward people with EDs. These findings are consistent with the contact hypothesis. This social psychology hypothesis, developed by Allport [99], argues that negative attitudes result from a lack of personal and positive contact with out-group members. This confirms the relevance of stigma reduction strategies that promote interpersonal or intergroup interactions with stigmatized people. A few studies included in this review highlighted the impact of other sociodemographic variables on the extent of ED stigma, such as a person’s ethnic group, weight category, and degree program. Future research would be helpful to replicate the impact of these variables on ED stigma.

Finally, the results on stigma distribution lead us to wonder what individual characteristics would protect one from ED stigma and self-stigma. Evans-Lacko et al. [100] found that having a university education or being employed resulted in less self-stigma about mental illnesses and more empowerment, as well as finding that women felt less empowered and perceived more mental illness discrimination. However, no specific work has been conducted regarding ED stigma.

Our review revealed that very few studies have focused on the *consequences* of ED stigma. Foran et al. [4] reviewed 9 studies on this dimension, and this present review examines 10; 7 of these studies are common to both works. The difference for the remaining texts can be explained by different choices in terms of keywords and inclusion criteria between our reviews. Nevertheless, despite these differences, our present review synthesizes similar negative impacts of ED stigma to those identified by Foran et al. [4]; namely, ED stigma precipitated negative emotions and decreased self-esteem in people with EDs, which is notable as these symptoms are already prevalent in EDs. Moreover, ED stigma exacerbated ED symptoms, specifically inducing more inappropriate eating behaviors and delaying treatment-seeking. Indeed, a recent meta-analysis [101] found a small-to-medium association between various combined discriminations (i.e., general, racial/ethnic, gender, LGBT, weight/obesity, and sexual harassment) and ED symptoms (e.g., general ED symptoms, body dissatisfaction, binge eating, restraint, night eating, etc.). The data of this meta-analysis and the present review are worrying because they indicate that discrimination increases the risk of developing an ED or maintaining an existing ED. Finally, these data raise questions about the impact of stigma on the eating behavior of people with multiple devalued attributes, such as a black woman with AN, but this topic seems still under-investigated.

In addition, people with EDs were notably aware of ED stigma. Stigmatization may therefore lead to self-stigma (e.g., people with EDs feeling they are responsible for their condition), which has been linked to negative emotions and reduced treatment-seeking. In short, ED stigma may lock people with EDs into a vicious circle that decreases the likelihood of recovery. The literature on mental illness has also demonstrated self-stigma among patients with mental illnesses [18]. If we apply the stage model of self-stigma developed by Corrigan and Rao [102] to the current evidence review, it would seem that people with AN have moved beyond the stage of “awareness” and “application”, and into “appropriation”. Future research should address whether people with AN might have reached the advanced level of “harm”; at that stage, individuals lack a sense of capability, which interferes with their life-goal achievement. As much of the research has focused on the perceptions of people with AN, it would be beneficial to assess self-stigma stages in people with BN and BED. An enhanced understanding of the self-stigma process in ED would enable more effective mitigation actions. Regarding mental illness, Rüsch et al. [103] have identified a range of factors associated with the emergence of self-stigma: the perception of others’ negative responses, belief in the legitimacy of others’ reactions, and a high degree of group identification. It would be interesting to establish whether these and other potential factors play a role in ED self-stigma.

### 4.2. Challenges and Opportunities in the Study of ED Stigma

Firstly, this review highlights a lack of diversity in methodological tools in the body of research on ED stigma. All of the studies included in this review exclusively used explicit measures. The use of such transparent measures to assess ED stigma may have elicited social desirability bias in study participants. This tendency to cast oneself, consciously or not, in a positive light, or present oneself in agreement with social expectations [104], may have motivated participants to report fewer stigmatizing behaviors and attitudes, leading to an underestimation of ED stigma prevalence. However, the two studies in this review that explicitly assessed social desirability effects found no significant association between participants’ “inclination to social desirability” and their stigmatizing attitudes [1,68]. Nevertheless, future research should include implicit measures to counteract the potential confounding effects of bias in the evaluation of ED stigma. Research on mental illness stigma has generally included both implicit attitude measures (e.g., a go/no-go task) and self-report measures [105,106,107,108,109]. Indeed, implicit and explicit attitude measures are not necessarily correlated [105,106], but could complement one another. Sometimes both types of attitudes are negative [107], but in other cases, only explicit [106] or implicit attitudes are negative [108]. Moreover, implicit and explicit attitudes do not necessarily predict the same consequences. In their study on decision patterns in healthcare professionals, Peris et al. [105] found that explicit negative attitudes significantly predicted more unfavorable prognoses for patients, while implicit attitudes were predictive of overdiagnosis (i.e., adding comorbidities). Teachman et al. [107] observed that in-group members possessed an implicit and explicit negative bias toward people with a mental illness. Future studies on ED self-stigma could likewise include implicit assessments [110].

Secondly, the review points to unequal research efforts in ED stigma, as the majority of studies focused on AN. The lack of interest in BED is one of the major limitations of the reviewed body of research. The recent inclusion of this disorder in the *DSM-5* as a specific ED may explain this deficit.

Additionally, despite finding evidence of the harmful effects of ED stigma, this review reveals that very few studies to date have examined the consequences of ED stigma. The rare studies that have addressed this issue suggest that ED stigma adversely affects the medical treatment of people with ED. Further research is needed, in particular, to elucidate the mechanism that links stigma with treatment avoidance. The aim would be to establish whether stigma impedes treatment-seeking, and the extent to which people with ED might use stigma to rationalize treatment avoidance.

Finally, one included study highlighted that patients’ recovery attitude decreased when they perceived that their family members also experienced stigma. Apart from this study, we are not aware of any studies that have reported family stigma in the field of EDs. However, studies conducted on people (e.g., parents, sibling, etc.) who had a family member with a mental health issue found that they reported stigma experiences through their association with that family member [44,45]. Relatives reported experiences of overt stigma: blame, rejection, avoidance, embarrassment, verbal abuse, loss of status, and name-calling. They also reported psychological (i.e., stress, anxiety, lower self-esteem), social (i.e., avoiding situations, hiding their relative’s situation), and family consequences (i.e., seeing the relative as a burden, being stigmatizing) [44,45]. Considering these data, family stigma deserves special attention in the field of EDs. Research is needed to detect ED family stigma and its consequences on relatives (e.g., risk of developing their own mental issue) and patients themselves (e.g., shame, culpability, avoidance of treatment-seeking).

### 4.3. Clinical Implications

Considering the potential consequences of ED stigma, more work is urgently needed to reduce stigma. The review has highlighted that being familiar with or knowledgeable about EDs is associated with less ED stigma [25,26,32,46,47,60]. Therefore, improving public awareness about EDs may be an effective strategy to reduce ED stigma. Research on mental illness stigma has demonstrated that anti-stigma educational interventions and facilitating contact with people experiencing mental illnesses leads, in the short term, to knowledge gains and positive attitude changes [103,111,112,113]. However, evidence from the few programs specifically targeting ED stigma suggests that due consideration should be given to the exact messaging used in interventions. Reinforcing causal explanations for ED that focus on sociocultural influences could exacerbate stigmatizing attitudes [57,114,115,116,117]. Further work is required to determine the factors that contribute to public ED stigma in order to design suitable interventions that mitigate ED stigma and avoid disseminating information that could reinforce it.

The present review has revealed that each ED possesses unique stigmatizing content, and that some populations are more stigmatizing toward people with EDs (e.g., men) than others (e.g., psychology students). Thus, it also seems essential to tailor the intervention content to the specific ED, and adapt the design to the target audience. The existing research on mental illness stigma illustrates the utility of adapting strategies to particular groups. In their review, Thornicroft et al. [112] determined that adults were more responsive to education, while adolescents benefited from direct contact with stigmatized individuals. Our review indicates that young people are one of the most stigmatizing groups toward EDs. We hypothesize that contact-focused strategies would be the preferred choice to mitigate ED stigma in the least tolerant groups. Virtual and augmented reality (i.e., VR and AR) technologies offer a novel mechanism to simulate interpersonal and intergroup contact between stigmatizing groups and people with EDs. In this way, Christofi and Michael-Grigoriou [118] have reported promising effects of full-body VR illusion on empathy and prejudice toward minority people (e.g., black people or people with a disability).

Notably, while there is a clear need to reduce ED stigma in the general population, there is also a need to inform healthcare professionals who were found to hold similarly negative beliefs and attitudes toward people with EDs [3,46,49,53,55,116]. The effects of anti-stigma interventions for mental health professionals have received little attention to date [103,112]. Thornicroft et al. [112] reported positive attitude changes in practitioners, but these benefits did not necessarily translate to improved experiences for people with mental illness. As a first step toward designing effective anti-stigma programs for the medical community, future research should focus on identifying which comments, attitudes, or behaviors of healthcare professional patients experience as stigmatizing. More work is also needed to evaluate the effectiveness of interventions among healthcare professionals, and the indirect effects on patients’ motivation to engage in a caring process.

Finally, the review has demonstrated that, along with stigma, some people with EDs self-stigmatize. People with EDs must be protected from the potential additional harm of self-stigma. Programs based on cognitive behavioral therapies, acceptance and commitment therapies, psychoeducation, mindfulness, and peer support are already being used to effectively reduce self-stigma in people experiencing mental illness [119,120]. Future studies should evaluate the effectiveness and replicability of these interventions on self-stigma in people with EDs. Similarly, if there is evidence of family stigma among the relatives of patients with EDs, these different therapies should be proposed to help them cope. Indeed, a recent study showed that emotion-focused family therapy decreased parental fear and parental self-blame, consequently enhancing parental self-efficacy [121]. Moreover, this improvement in self-efficacy increased parental intentions to engage with supportive efforts in recovery and emotional coaching behaviors. If this therapy is useful for dealing with self-blame, perhaps it will be useful for dealing with the blame of others.

### 4.4. Limitations

Because our literature search was subject to some inclusion criteria (e.g., year of publication) and restricted to four major databases in psychology, this review on ED stigma might not be exhaustive. Indeed, a different choice of databases (e.g., Scopus or Web of Science) might lead to different results. Similarly, the choice of keywords may have reduced the number of studies included in the review process.

In addition, it should be noted that only the first author conducted this review process. Therefore, although we have clearly stated objective inclusion/exclusion criteria, some subjectivity may reside in this work, which may weaken its scientific value. However, it should be noted that in the event of uncertainty regarding the inclusion of an article, a second researcher (V.F.) was consulted.

Finally, we used a qualitative review approach, and the quality of each study was not assessed as part of the search process. Therefore, this review may include articles with methodological and other biases. A quantitative meta-analysis might be helpful to provide additional and novel insights. Because we did not restrict our inclusion criteria to particular methodological designs, the (presumed) methodological heterogeneity would hinder statistical comparisons, but meta-analysis could be used for subgroups of studies. This heterogeneity in the studies’ methods may also have constrained the conclusions that this review could draw. However, this variability is also emblematic of the recent interest in this topic, and indicates diverse engagement in ED stigma research.

## 5. Conclusions

This scoping review provides some evidence of ED stigma in the general public, as well as among healthcare professionals; it also highlights the harmful impacts of ED stigma on the psychological state (e.g., low self-esteem, self-stigma) and treatment-seeking behaviors of people with EDs. Educating the public and healthcare professionals on ED stigma seems necessary to avoid missed or delayed diagnosis and improve the care of patients with EDs.

Moreover, the review highlights the underrepresentation of research on BN and BED compared to the interest in AN, while also noting that stigma content differs between the three ED disorders (specifically for BED). The consequences of ED stigma are poorly studied and understood relative to ED content and distribution. Thus, more research is required to provide insight into ED stigma and improve the diagnosis and treatment of people with EDs.

## Figures and Tables

**Table 1:** Summary of studies included in the scoping review.

Reference	Location	Final Sample	Study Design	ED Concerned	ED Stigma
[29]	USA	91 Volunteers	Vignette paradigm and questionnaire completion	AN	Content
[32]	Australia	135 College students (only women)	Vignette paradigm and questionnaire completion	AN	Content
[28]	USA	102 Psychology students (only women)	Vignette paradigm and questionnaire completion	AN	Content
[49]	USA	148 Healthcare professionals	Vignette paradigm and questionnaire completion	BN	Content
[50]	USA	360 Psychology students	Vignette paradigm and questionnaire completion	BN	Content
[51]	Canada	421 Adult volunteers	Vignette paradigm and questionnaire completion	BED	Content
[52]	USA	118 Psychology students	Vignette paradigm	AN + BN	Content
[53]	USA	82 Pediatric residents and nurses	Questionnaire completion	AN + BN	Content
[1]	USA	447 Psychology students	Vignette paradigm and questionnaire completion	All EDs	Content
[54]	USA	318 College students	Vignette paradigm and questionnaire completion	All EDs	Content
[55]	Singapore	19 Nurses	Questionnaire completion	All EDs	Content
[2]	Germany	729: 267 college students and 523 nonstudents	Vignette paradigm and questionnaire completion	All EDs	Content
[56]	Turkey	49 school counselor volunteers	Vignette paradigm and questionnaire completion	All EDs	Content
[30]	UK	125 College students (only women)	Vignette paradigm and questionnaire completion	AN	Content Distribution
[57]	USA	80 College students (only women)	Vignette paradigm and questionnaire completion	AN	Content Distribution
[58]	Australia	343 Psychology students	Vignette paradigm and questionnaire completion	AN	Content Distribution
[59]	Ireland, UK	152 Sport-based professionals	Questionnaire completion	AN	Content Distribution
[27]	USA	86 Psychology students	Vignette paradigm and questionnaire completion	AN	Content Distribution
[60]	Australia	208 Australian voters	Vignette paradigm, semi-structured interview, and questionnaire completion	BN	Content Distribution
[61]	Australia	208 Australian voters	Vignette paradigm, semi-structured interview, and questionnaire completion	BN	Content Distribution
[31]	Australia	1828 Australian voters	Questionnaire completion	BN	Content Distribution
[47]	Australia	1828 Australian voters	Questionnaire completion	BN	Content Distribution
[25]	Italia	2109 College students (<30 years)	Questionnaire completion	AN + BN	Content Distribution
[46]	USA	80 Physicians	Questionnaire completion	AN + BN	Content Distribution
[26]	Italia	517 Nursing students	Questionnaire completion	AN + BN	Content Distribution
[33]	USA	1447 Psychology students	Vignette paradigm and questionnaire completion	AN + BED	Content Distribution
[62]	Australia	1135 Adolescent volunteers	Vignette paradigm and questionnaire completion	BN + BED	Content Distribution
[3]	Ireland	171 Healthcare professionals	Vignette paradigm and questionnaire completion	All EDs	Content Distribution
[63]	Ireland	283 Adolescent volunteers	Vignette paradigm and questionnaire completion	All EDs	Content Distribution
[7]	Australia	126 Psychology and physician students	Questionnaire completion	AN	Distribution
[64]	UK	~3500 adult volunteers	Unknown	AN + BN	Distribution
[65]	Australia	402 College students	Vignette paradigm	AN + BN	Distribution
[66]	USA	235 Psychology students	Vignette paradigm and questionnaire completion	AN + BN	Distribution
[67]	Germany	2014 Adult volunteers	Interview and vignette paradigm	AN + BN	Distribution
[68]	USA	447 Psychology students	Vignette paradigm and questionnaire completion	All EDs	Distribution
[8]	Australia	545 Psychology students	Vignette paradigm and questionnaire completion	All EDs	Distribution
[6]	Germany	36 Adolescent patients with EDs (only women)	Questionnaire completion	AN	Consequences
[69]	Canada	19 Patients with EDs (only women)	Semi-structured interview	AN	Consequences
[42]	Canada	36 Patients with EDs (only women)	Questionnaire completion	AN	consequences
[70]	Norway	38 patients with EDs (only women)	Semi-structured interview	BN	Consequences
[71]	Australia	35 Adult volunteers with EDs (only women)	Semi-structured interview	AN + BN	Consequences
[72]	USA	145 Psychology students with EDs	Questionnaire completion	All EDs	Consequences
[73]	Australia	452 Adult volunteers with EDS	Questionnaire completion	All EDs	Consequences
[5]	Australia, USA, UK	317 Adult volunteers with EDs	Questionnaire completion	All EDs	Consequences
[74]	Australia, USA, UK	485 Adult volunteers with EDs (diagnosed and undiagnosed)	Questionnaire completion	All EDs	Consequences
[75]	Australia, USA, UK	260 Adult volunteers with EDs	Questionnaire completion	All EDs	Consequences

Note: EDs: eating disorders; AN: anorexia nervosa; BN: bulimia nervosa; BED: binge eating disorder.

**Table 2:** Distribution of ED stigma.

Variable	Total Number of Studies	Target(s)	Main Results		Reference
			Most Stigmatizing Group	Least Stigmatizing Group	
Gender	14	EDs/AN/BN/BED	Men	Women	[7,8,25,26,27,31,33,47,58,59,62,65,66,67]
Familiarity with EDs	8	EDs/AN/BN	Unfamiliar people	Familiar people	[25,26,27,32,46,47,60,61]
Age	4	AN/BN	Young adults	Old adults	[25,31,61,64]
Education	4	AN/BN	People with low education	People with high education	[31,60,61,64]
Symptoms of EDs	2	AN	People with a low level of symptoms	People with a high level of symptoms	[30,66]
Degree program	2	EDs/AN	Medicine, sociology, education, science, economics, law, and statistics students	Psychology students	[7,25]
Income	2	AN/BN	People with low income	People with high income	[31,64]
Weight category	1	AN/BN	Normal-weight and overweight people	Underweight people	[25]
Ethnicity	1	EDs	African Americans	Caucasian Americans	[66]
Information/knowledge about EDs	1	EDs	Uninformed people	Informed people	[47]
Just world belief	1	EDs	Believers	Non-believers	[68]

Note: EDs: eating disorders; AN: anorexia nervosa; BN: bulimia nervosa; BED: binge eating disorder.

## Data Availability

Not applicable.

## Appendix A

**Table A1:** Details of the tools used in the included studies.

Reference	Tools
[29]	Vignette paradigm Twenty-item characteristics scale Level of interpersonal discomfort scale Opinion scale
[32]	Vignette paradigm Affective reaction scale Social distance scale Causal attribution
[28]	Vignette paradigm Items of: Biological attribution scale Vanity attitude scale Self-responsibility attribution scale Admiration reaction scale Sympathy reaction scale Anger reaction scale Coercion into treatment scale Imitation scale Social distance scale
[49]	Vignette paradigm Semantic differential scale
[50]	Vignette paradigm Items of the attributional model
[51]	Vignette paradigm Characteristics scale Affective reactions scale and social distance scale Blame attribution scale Balanced inventory of desirable responding Item for assumptions about weight status associated with BED
[52]	Vignette paradigm
[53]	Items created by authors
[1]	Vignette paradigm Universal stigma scale Marlowe–Crowne Social Desirability Scale
[54]	Vignette paradigm Twenty-item universal measure of bias Eleven-item universal stigma Sscale
[55]	Items based on *DSM-5* diagnostic criteria Items based on Crisp et al., 2000; Currin, Waller, and Schmidt, 2009; Green, Johnston, Cabrini, Fornai, and Kendrick, 2008; Hay et al., 2007; Jones, Saeidi, and Morgan, 2012; NCCMH, 2004
[2]	Vignette paradigm Universal stigma scale Item from Ebneter and Latner (2013) for lack of self-discipline German version (Angermeyer and Matschinger, 2005) of the seven-item scale developed by Link et al. (1987) for desire for social distance
[56]	Vignette paradigm Twelve-item illness perceptions questionnaire Item for social distance
[30]	Vignette paradigm Measures based on Jorm et al. (1999); Crisp et al. (2000); Corrigan et al. (2003); Mond et al. (2004a) Level of familiarity questionnaire SCOFF questionnaire (Sick-Control-One Stone-Fat-Food)
[57]	Vignette paradigm Items of: Opinions scale Characteristics scale Affective reaction scale Social distance scale Level of interpersonal discomfort scale Perception of community norms Measure of personal acquaintance
[58]	Vignette paradigm Measures created by Crisp et al. (2000), Mond et al. (2006); Roehrig and McLean (2010); Stewart et al. (2006)
[59]	Items of the opinions scale Items of the international research on personal stigma
[27]	Vignette paradigm Items of Dejong 1980; Heblet and Mannix, 2003
[60]	Eating disorder examination questionnaire (EDE-Q) General psychological distress (K10) Medical outcome study short form (SF-12) Vignette paradigm Semi-structured interview
[61]	Vignette paradigm Semi-structured interview
[31]	Stigmatizing attitudes and beliefs about BN
[47]	Stigmatizing attitudes and beliefs about BN Level of familiarity questionnaire Eating disorder examination questionnaire (EDE-Q)
[25]	Stigmatizing attitudes and beliefs about BN Stigmatizing attitudes and beliefs about AN
[46]	Items adapted from Currin et al. 2009 Items derived from the illness perception questionnaire
[26]	Italian version of the McLean SAB-BN-ITA for BN and adapted for AN
[33]	Vignette paradigm Universal measure of bias Universal stigma scale Items for psychopathology perceived based on previous works.
[62]	Vignette paradigm Items based on perceptions of severity, Australian Bureau of Statistics, 2011; Mond and Arrighi 2011; Mond et al. 2004
[3]	Vignette paradigm Illness perceptions questionnaire
[63]	Vignette paradigm Illness perceptions questionnaire Affective reaction and personality characteristics, adapted from Penn et al. (1994)
[7]	Causal attributions scale Opinions scale Eating disorder stigma scale Characteristics scale Affective reaction scale
[64]	Unknow
[65]	Vignette paradigm
[66]	Levels of contact report Vignette paradigm Attribute rating scale Eating attitude test
[67]	Interview Vignette paradigm
[68]	Vignette paradigm Depression stigma scale Opinion scale Just world scale Marlowe–Crowne Social Desirability Scale
[8]	Masculine norms inventory 46 Conformity to feminine norms inventory 45 Vignette paradigm Items created by Crisp et al., 2000; Ebneter and Latner 2013; Griffiths et al. 2015; Roehrig and McLean 2010; Stewart et al. 2006 Modified version of the level of contact scale Twelve-item eating disorder examination questionnaire
[6]	Items of: Devaluation–discrimination scale The consumers’ experience of stigma questionnaire Opinions scale Revised illness perception questionnaire
[69]	Semi-structured interview
[42]	Eating disorder examination questionnaire (EDE-Q) Devaluation of consumer scale Devaluation of consumer families scale Internalized stigma of mental illness Rosenberg Self-Esteem Scale General self-efficacy scale Recovery assessment scale
[70]	Semi-structured interview
[71]	Semi-structured interview based on Darcy et al. 2010; Easter, 2012
[72]	Eating attitude test (EAT-26) Self-stigma seeking help scale Attitude toward seeking professional psychological help—short gorm Disclosure expectations scale
[73]	Five-subscale internalized stigma of mental illness scale Eating disorder examination questionnaire (EDE-Q) Self-stigma of seeking help scale Twenty-one-item depression anxiety stress scale
[5]	Self-stigma of seeking help scale Twenty-one-item depression anxiety stress scale Ten-item self-esteem scale Eating disorder examination questionnaire (EDE-Q)
[74]	Self-stigma of seeking help scale Perceived discrimination and devaluation scale Eating disorder examination questionnaire (EDE-Q)
[75]	Eating disorder examination questionnaire (EDE-Q) Discrimination exposure subscale of the internalized stigma of mental illness scale Alienation subscale of the modified internalized stigma of mental illness scale (ISMI) Social withdrawal subscale of the modified ISMI

## Appendix B

**Table A2:** Overview of stigma content for each eating disorder.

	Anorexia Nervosa (AN)	Bulimia Nervosa (BN)	Binge Eating Disorder (BED)
STEREOTYPES			
Responsibility	Responsible for their disorder, to blame for their disorder		
Character traits	Self-destructive, dangerous, incompetent, able to pull themselves together, difficult to talk to, attention-seeking	Self-destructive	Larger body, less desirable personality traits than non-BED individuals,
Gender attribution	Women	Inconclusive	Men (non-significant)
Disorder severity and control	Not a very severe disorder, low level of minimization, low perception of benefits	Little minimization, little perception of benefits, a severe disorder, difficult to treat	
Supposed causes Internal	Lack of self-discipline, desire for attention, vanity	Lacking self-discipline, having low self-esteem	Lacking self-discipline, self-control, and willpower
External	Lacking social support and parental support, sociocultural influences (media).	Lacking social support and parental support, sexual abuse, overweight/obesity during childhood/adolescence, sociocultural influences (media).	Lacking social support
PREJUDICE			
Emotions involved	Desire to imitate, admiration, attractiveness, irritation, anger, little sympathy, discomfort in interaction	Desire to imitate, sympathy	Negative reactions, greater prejudice for BED than for non-BED individuals
DISCRIMINATION			
Behaviors involved	Social distance, reluctance to offer work	Social Distance	Social distance (but not always observed)
COMPARISON BETWEEN EDs			
	More self-discipline than BN/BED, More distrustful than BED	More to blame for their disorder, more lack of discipline, more self-destructive, less admirable, and less desire for social distance than AN, More severe and more distrustful than BED	More responsible, lacking willpower/self-discipline/control, more negative reactions than AN/BN, but perceived as more attractive and associated with fewer negative character traits and less desire for social distance. No attribution to poor parental support
COMPARISON WITH OTHER MENTAL DISORDERS			
	More responsible but less control, distrust, and desire for social distance than depression or MDE; a longer illness than depression; fewer positive reactions		
	More negative assessment; Less intelligent, more driven, more disciplined, more enthusiastic, and less lazy than depression, evoking more anger but also more admiration; Greater lack of social support, parental support, and self-discipline than schizophrenia, but perceived as less dangerous, more able to pull themselves together, their condition is less likely to be attributed to genetic and biological factors, and people feel less discomfort in interaction; More perceived as a psychopathology than ARFID and other eating problems	More friendly attitudes than depression	More negative assessment; More personal control and less reliable than depression; More perceived as a psychopathology than ARFID and other eating problems; More negative traits and more blame for obesity without BED than obesity with BED
COMPARISON WITH OTHER PHYSICAL DISORDERS			
	Less responsibility but more distrust and more desire for social distance than obesity; more negative assessment; fewer positive reactions		
	More able to pull themselves together than asthma; Greater lack of social support, parental support, and self-discipline than asthma and mononucleosis; Condition less likely to be attributed to genetic and biological factors than asthma, mononucleosis, obesity, and skin cancer; More social distance than obesity and skin cancer		More personal control than type 1 diabetes
HEALTHCARE PROFESSIONALS			
Beliefs	Responsible for their condition, manipulative, disrespectful, deceitful, non-compliant with treatment; Fear, stress, anger, exasperation, displeasure, discomfort, frustration, preference to treat other patients, decreased motivation to treat		
	Unreliable		
Emotions/behaviors	More reluctance to manage than type 1 diabetes	More negative reaction than cocaine users and healthy athletes	

Please refer to Section 3.2. “Content of Stigma” in the main text to find relevant references for each cell. Note: ARFID: avoidant restrictive food intake disorder; MDE: major depressive episode.

## References

[1] Ebneter D.S., Latner J.D. (2013). Stigmatizing attitudes differ across mental health disorders: A comparison of stigma across eating disorders, obesity, and major depressive disorder. J. Nerv. Ment. Dis..

[2] Thörel N., Thörel E., Tuschen-Caffier B. (2021). Differential stigmatization in the context of eating disorders: Less blame might come at the price of greater social rejection. Stigma. Health.

[3] McNicholas F., O’Connor C., O’Hara L., McNamara N. (2016). Stigma and treatment of eating disorders in Ireland: Healthcare professionals’ knowledge and attitudes. Ir. J. Psychol. Med..

[4] Foran A.M., O’Donnell A.T., Muldoon O.T. (2020). Stigma of eating disorders and recovery-related outcomes: A systematic review. Eur. Eat. Disord. Rev..

[5] Griffiths S., Mond J.M., Murray S.B., Touyz S. (2015). The prevalence and adverse associations of stigmatization in people with eating disorders. Int. J. Eat. Disord..

[6] Maier A., Ernst J.P., Müller S., Gross D., Zepf F.D., Herpertz-Dahlmann B., Hagenah U. (2014). Self-perceived stigmatization in female patients with anorexia nervosa—Results from an explorative retrospective pilot study of adolescents. Psychopathology.

[7] Bannatyne A.J., Stapleton P.B. (2016). Attitudes towards anorexia nervosa: Volitional stigma differences in a sample of pre-clinical medicine and psychology students. J. Ment. Health.

[8] Austen E., Griffiths S. (2019). Why do men stigmatize individuals with eating disorders more than women? Experimental evidence that sex differences in conformity to gender norms, not biological sex, drive eating disorders’ stigmatization. Eat. Disord. J. Treat. Prev..

[9] Bucchianeri M.M., Eisenberg M.E., Neumark-Sztainer D. (2013). Weightism, Racism, Classism, and Sexism: Shared Forms of Harassment in Adolescents. J. Adolesc. Health.

[10] Jones K.P., Sabat I.E., King E.B., Ahmad A., McCausland T.C., Chen T. (2017). Isms and schisms: A meta-analysis of the prejudice-discrimination relationship across racism, sexism, and ageism. J. Organ. Behav..

[11] Goffman E., Prentice H. (1963). Stigma: Notes on the Management of Spoiled Identity.

[12] Angermeyer M.C., Dietrich S. (2006). Public beliefs about and attitudes towards people with mental illness: A review of population studies. Acta Psychiatr. Scand..

[13] Crisp A.H., Gelder M.G., Rix S., Meltzer H.I., Rowlands O.J. (2000). Stigmatisation of people with mental illnesses. Br. J. Psychiatry.

[14] Kowert R., Griffiths M.D., Oldmeadow J.A. (2012). Geek or Chic? Emerging Stereotypes of Online Gamers. Bull Sci. Technol. Soc..

[15] Vartanian L.R. (2010). Disgust and perceived control in attitudes toward obese people. Int. J. Obes..

[16] Al Ramiah A., Hewstone M., Dovidio J.F., Penner L.A., Russell H., Bond L., McGinnity F. (2010). The social psychology of discrimination: Theory, measurement, and consequences. Making Equality Count: Irish and International Approaches to Measuring Discrimination.

[17] Bertrand M., Mullainathan S. (2003). Are emily and greg more employable than lakisha and jamal? a field experiment on labor market discrimination. NBER Work. Pap..

[18] Corrigan P.W., Rüsch N. (2002). Mental Illness Stereotypes and Clinical Care: Do People Avoid Treatment Because of Stigma?. Psychiatr. Rehabil. Ski..

[19] Corrigan P.W., Watson A.C. (2002). The paradox of self-stigma and mental illness. Clin. Psychol. Sci. Pract..

[20] Hipes C., Lucas J., Phelan J.C., White R.C. (2016). The stigma of mental illness in the labor market. Soc. Sci. Res..

[21] American Psychiatric Association (2013). Diagnostic and Statistical Manual of Mental Disorders.

[22] Puhl R., Suh Y. (2015). Stigma and Eating and Weight Disorders. Curr. Psychiatry Rep..

[23] Smith K.E., Ellison J.M., Crosby R.D., Engel S.G., Mitchell J.E., Crow S.J., Peterson C.B., Le Grange D., Wonderlich S.A. (2017). The validity of DSM-5 severity specifiers for anorexia nervosa, bulimia nervosa, and binge-eating disorder. Int. J. Eat..

[24] Ágh T., Kovács G., Supina D., Pawaskar M., Herman B.K., Vokó Z., Sheehan D.V. (2016). A systematic review of the health-related quality of life and economic burdens of anorexia nervosa, bulimia nervosa, and binge eating disorder. Eat. Weight. Disord..

[25] Caslini M., Crocamo C., Dakanalis A., Tremolada M., Clerici M., Carrà G. (2016). Stigmatizing attitudes and beliefs about anorexia and bulimia nervosa among Italian undergraduates. J. Nerv. Ment. Dis..

[26] Lupo R., Zaminga M., Carriero M.C., Santoro P., Artioli G., Calabrò A., Ilari F., Benedetto A., Caslini M., Clerici M. (2020). “Eating disorders and related stigma”: Analysis among a population of Italian nursing students. Acta Biomed..

[27] Varnado-Sullivan P.J., Parker C.C., Rohner A. (2020). Stigmatization and knowledge of anorexia nervosa. Eat. Weight. Disord..

[28] Geerling D.M., Saunders S.M. (2015). College students’ perceptions of individuals with anorexia nervosa: Irritation and admiration. J. Ment. Health.

[29] Stewart M.-C., Keel P.K., Schiavo R.S. (2006). Stigmatization of Anorexia Nervosa. Int. J. Eat. Disord..

[30] Mond J.M., Robertson-Smith G., Vetere A. (2006). Stigma and eating disorders: Is there evidence of negative attitudes towards anorexia nervosa among women in the community?. J. Ment. Health.

[31] McLean S.A., Paxton S.J., Massey R., Hay P.J., Mond J.M., Rodgers B. (2014). Stigmatizing attitudes and beliefs about bulimia nervosa: Gender, age, education and income variability in a community sample. Int. J. Eat. Disord..

[32] Zwickert K., Rieger E. (2013). Stigmatizing attitudes towards individuals with anorexia nervosa: An investigation of attribution theory. J. Eat. Disord..

[33] Ellis J.M., Essayli J.H., Zickgraf H.F., Rossi J., Hlavka R., Carels R.A., Whited M.C. (2020). Comparing stigmatizing attitudes toward anorexia nervosa, binge-eating disorder, avoidant-restrictive food intake disorder, and subthreshold eating behaviors in college students. Eat. Behav..

[34] Thompson-Brenner H., Satir D.A., Franko D.L., Herzog D.B. (2012). Clinician Reactions to Patients With Eating Disorders: A review of the Literature. Psychiatr. Serv..

[35] Rome E.S., Ammerman S. (2003). Medical complications of eating disorders: An update. J. Adolesc. Health.

[36] Hart L.M., Granillo M.T., Jorm A.F., Paxton S.J. (2011). Unmet need for treatment in the eating disorders: A systematic review of eating disorder specific treatment seeking among community cases. Clin. Psychol. Rev..

[37] Brewis A.A. (2014). Social Science & Medicine Stigma and the perpetuation of obesity. Soc. Sci. Med..

[38] Brochu P.M., Esses V.M. (2011). What’s in a Name? The Effects of the Labels “Fat” Versus “Overweight” on Weight Bias. J. Appl. Soc. Psychol..

[39] Lewis S., Thomas S.L., Blood R.W., Castle D.J., Hyde J., Komesaroff P.A. (2011). How do obese individuals perceive and respond to the different types of obesity stigma that they encounter in their daily lives? A qualitative study. Soc. Sci. Med..

[40] Vartanian L.R., Shaprow J.G. (2008). Effects of weight stigma on exercise motivation and behavior: A preliminary investigation among college-aged females. J. Health Psychol..

[41] Vartanian L.R., Porter A.M. (2016). Weight stigma and eating behavior: A review of the literature. Appetite.

[42] Dimitropoulos G., Mccallum L., Colasanto M., Freeman V.E., Gadalla T. (2016). The effects of stigma on recovery attitudes in people with anorexia nervosa in intensive treatment. J. Nerv. Ment. Dis..

[43] Tricco A.C., Lillie E., Zarin W., O’Brien K.K., Colquhoun H., Levac D., Moher D., Peters M.D.J., Horsley T., Weeks L. (2018). PRISMA extension for scoping reviews (PRISMA-ScR): Checklist and explanation. Ann. Intern. Med..

[44] Larson J.E., Corrigan P. (2008). The Stigma of Families with Mental Illness. Acad. Psychiatry.

[45] Liegghio M. (2017). ‘Not a good person’: Family stigma of mental illness from the perspectives of young siblings. Child. Fam. Soc. Work.

[46] Anderson K., Accurso E.C., Kinasz K.R., Le Grange D. (2016). Residents’ and Fellows’ Knowledge and Attitudes about Eating Disorders at an Academic Medical Center. Acad. Psychiatry.

[47] Rodgers R.F., Paxton S.J., McLean S.A., Massey R., Mond J.M., Hay P.J., Rodgers B. (2015). Stigmatizing attitudes and beliefs toward bulimia nervosa: The importance of knowledge and eating disorder symptoms. J. Nerv. Ment. Dis..

[48] Furnham A. (2003). Belief in a just world: Research progress over the past decade. Pers. Individ. Dif..

[49] Yu J., Hildebrandt T., Lanzieri N. (2015). Healthcare professionals’ stigmatization of men with anabolic androgenic steroid use and eating disorders. Body Image..

[50] Vaughn A.A., Lowe J.D. (2020). With age comes responsibility: Changes in stigma for boys/men with bulimia nervosa. Eat. Weight Disord..

[51] Hollett K.B., Carter J.C. (2021). Separating Binge Eating Disorder Stigma and Weight Stigma: A Vignette Study. Int. J. Eat. Disord..

[52] Roehrig J.P., McLean C.P. (2010). A comparison of stigma toward eating disorders versus depression. Int. J. Eat. Disord..

[53] Raveneau G., Feinstein R., Rosen L.M., Fisher M. (2014). Attitudes and knowledge levels of nurses and residents caring for adolescents with an eating disorder. Int. J. Adolesc. Med. Health.

[54] Murakami J.M., Essayli J.H., Latner J.D. (2016). The relative stigmatization of eating disorders and obesity in males and females. Appetite.

[55] Seah X.Y., Tham X.C., Kamaruzaman N.R., Yobas P.K. (2018). Nurses’ perception of knowledge, attitudes and reported practice towards patients with eating disorders: A concurrent mixed-methods study. Arch. Psychiatr. Nurs..

[56] Ogutlu H., McNicholas F. (2021). Stigma and Treatment of Eating Disorders in School Counselors in Turkey (STED-SCIT). Psychiatry Behav. Sci..

[57] Stewart M.-C., Schiavo R.S., Herzog D.B., Franko D.L. (2008). Stereotypes, Prejudice and Discrimination of Women with Anorexia Nervosa. Eur. Eat. Disord. Rev..

[58] Griffiths S., Mond J.M., Murray S.B., Touyz S. (2014). Young peoples’ stigmatizing attitudes and beliefs about anorexia nervosa and muscle dysmorphia. Int. J. Eat. Disord..

[59] McArdle S., Meade M.M., Burrows E. (2018). Service providers’ attitudes toward athletes with eating disorders. Clin. J. Sport. Med..

[60] Mond J.M., Hay P.J., Rodgers B., Owen C., Beumont P.J.V. (2004). Beliefs of women concerning the severity and prevalence of bulimia nervosa. Soc. Psychiatry Psychiatr. Epidemiol..

[61] Mond J.M., Hay P.J., Rodgers B., Owen C., Beumont P.J.V. (2004). Beliefs of women concerning causes and risk factors for bulimia nervosa. Aust. N. Z. J Psychiatry.

[62] Anderson R., Gratwick-Sarll K., Bentley C., Harrison C., Mond J. (2015). Adolescents perception of the severity of binge eating disorder: A population-based study. J. Ment. Health.

[63] O’Connor C., McNamara N., O’Hara L., McNicholas F. (2016). Eating disorder literacy and stigmatising attitudes towards anorexia, bulimia and binge eating disorder among adolescents. Adv. Eat. Disord. Theory Res. Pract..

[64] Crisp A.H. (2005). Stigmatization of and discrimination against people with eating disorders including a report of two nationwide surverys. Eur. Eat. Disord. Rev..

[65] Mond J.M., Arrighi A. (2011). Gender differences in perceptions of the severity and prevalence of eating disorders. Early Interv. Psychiatry.

[66] Wingfield N., Kelly N., Serdar K., Shivy V.A., Mazzeo S.E. (2011). College students’ perceptions of individuals with anorexia and bulimia nervosa. Int. J. Eat. Disord..

[67] Makowski A.C., Mnich E.E., Angermeyer M.C., Löwe B., Von dem Knesebeck O. (2015). Sex differences in attitudes towards females with eating disorders. Eat. Behav..

[68] Ebneter D.S., Latner J.D., O’Brien K.S. (2011). Just world beliefs, causal beliefs, and acquaintance: Associations with stigma toward eating disorders and obesity. Pers. Individ. Dif..

[69] Dimitropoulos G., Freeman V.E., Muskat S., Domingo A., McCallum L. (2015). You dont have anorexia, you just want to look like a celebrity": Perceived stigma in individuals with anorexia nervosa. J. Ment. Health.

[70] Pettersen G., Rosenvinge J.H., Ytterhus B. (2008). The “double life” of Bulimia: Patients’ experiences in daily life interactions. Eat. Disord. J. Treat. Prev..

[71] Bannatyne A.J., Stapleton P.B. (2016). Eating Disorder Patient Experiences of Volitional Stigma Within the Healthcare System and Views on Biogenetic Framing: A Qualitative Perspective. Aust. Psychol..

[72] Hackler A.H., Vogel D.L., Wade N.G. (2010). Attitudes Toward Seeking Professional Help for an Eating Disorder: The Role of stigma and anticipated outcomes. J. Couns. Dev..

[73] Griffiths S., Mond J.M., Murray S.B., Thornton C., Touyz S. (2015). Stigma resistance in eating disorders. Soc. Psychiatry Psychiatr. Epidemiol..

[74] Griffiths S., Mond J.M., Li Z., Gunatilake S., Murray S.B., Sheffield J., Touyz S. (2015). Self-stigma of seeking treatment and being male predict an increased likelihood of having an undiagnosed eating disorder. Int. J. Eat. Disord..

[75] Griffiths S., Mitchison D., Murray S.B., Mond J.M., Bastian B.B. (2018). How might eating disorders stigmatization worsen eating disorders symptom severity? Evaluation of a stigma internalization model. Int. J. Eat. Disord..

[76] Page M.J., McKenzie J.E., Bossuyt P.M., Boutron I., Hoffmann T.C., Mulrow C.D., Shamseer L., Tetzlaff J.M., Akl E.A., Brennan S.E. (2021). The PRISMA 2020 statement: An updated guideline for reporting systematic reviews. J. Clin. Epidemiol..

[77] Corrigan P.W., Watson A.C., Warpinski A.C., Gracia G. (2004). Implications of Educating the Public on Mental Illness, Violence, and Stigma. Psychiatr. Serv..

[78] Mukherjee R., Fialho A., Wijetunge A., Checinski K., Surgenor T. (2002). The stigmatisation of psychiatric illness: The attitudes of medical students and doctors in a London teaching hospital. Psychiatr. Bull.

[79] Larose J.G., Gorin A.A., Clarke M.M., Wing R.R. (2011). Beliefs about weight gain among young adults: Potential challenges to prevention. Obesity.

[80] Miller E., Halberstadt J. (2005). Media consumption, body image and thin ideals in New Zealand men and women. N. Z. J Psychol..

[81] Foster G.D., Wadden T.A., Makris A.P., Davidson D., Sanderson R.S., Allison D.B., Kessler A. (2003). Primary care physicians’ attitudes about obesity and its treatment. Obes. Res..

[82] Puhl R.M., Schwartz M.B., Brownell K.D. (2005). Impact of perceived consensus on stereotypes about obese people: A new approach for reducing bias. Health Psychol..

[83] Horsburgh-McLeod G., Latner J.D., O’Brien K.S. (2009). Unprompted generation of obesity stereotypes. Eat. Weight. Disord..

[84] Musher-Eizenman D.R., Holub S.C., Miller A.B., Goldstein S.E., Edwards-Leeper L. (2004). Body size stigmatization in preschool children: The role of control attributions. J. Pediatr. Psychol..

[85] Agerström J., Rooth D.O. (2011). The Role of Automatic Obesity Stereotypes in Real Hiring Discrimination. J. Appl. Psychol..

[86] Wott C.B., Carels R.A. (2010). Overt weight stigma, psychological distress and weight loss treatment outcomes. J. Health. Psychol..

[87] Ashmore J.A., Friedman K.E., Reichmann S.K., Musante G.J. (2008). Weight-based stigmatization, psychological distress, & binge eating behavior among obese treatment-seeking adults. Eat. Behav..

[88] Friedman K.E., Ashmore J.A., Applegate K.L. (2008). Recent experiences of weight-based stigmatization in a weight loss surgery population: Psychological and behavioral correlates. Obesity.

[89] Galbraith K., Elmquist J., White M.A., Grilo C.M., Lydecker J.A. (2019). Weighty decisions: How symptom severity and weight impact perceptions of bulimia nervosa. Int. J. Eat. Disord..

[90] Alfoukha M.M., Hamdan-Mansour A.M., Banihani M.A. (2019). Social and Psychological Factors Related to Risk of Eating Disorders Among High School Girls. J. Sch. Nurs..

[91] National Institute of Mental Health (2017). Eating Disorders Statistics Reports for the U.S.. https://www.nimh.nih.gov/health/statistics/eating-disorders.shtml.

[92] O’Hara S.K., Smith K.C. (2007). Presentation of eating disorders in the news media: What are the implications for patient diagnosis and treatment?. Patient Educ. Couns..

[93] Rao H., Mahadevappa H., Pillay P., Sessay M., Abraham A., Luty J. (2009). A study of stigmatized attitudes towards people with mental health problems among health professionals. J. Psychiatr. Ment. Health Nurs..

[94] Chambliss H.O., Finley C.E., Blair S.N. (2004). Attitudes toward Obese Individuals among Exercise Science Students. Med. Sci. Sports Exerc..

[95] Schwartz M.B., Chambliss H.O.N., Brownell K.D., Blair S.N., Billington C. (2003). Weight Bias among Health Professionals Specializing in Obesity. Obes. Res..

[96] Smink F.R.E., Van Hoeken D., Hoek H.W. (2012). Epidemiology of eating disorders: Incidence, prevalence and mortality rates. Curr. Psychiatry Rep..

[97] Arcelus J., Mitchell A.J., Wales J., Nielsen S. (2011). Mortality rates in patients with anorexia nervosa and other eating disorders: A meta-analysis of 36 studies. Arch. Gen. Psychiatry.

[98] Chesney E., Goodwin G.M., Fazel S. (2014). Risks of all-cause and suicide mortality in mental disorders: A meta-review. World Psychiatry.

[99] Allport G.W. (1954). Nature of Prejudice.

[100] Evans-Lacko S., Brohan E., Mojtabai R., Thornicroft G. (2012). Association between public views of mental illness and self-stigma among individuals with mental illness in 14 European countries. Psychol. Med..

[101] Mason T.B., Mozdzierz P., Wang S., Smith K.E. (2021). Discrimination and Eating Disorder Psychopathology: A Meta-Analysis. Behav. Ther..

[102] Corrigan P.W., Rao D. (2012). On the self-stigma of mental illness: Stages, disclosure, and strategies for change. Can. J. Psychiatry.

[103] Rüsch N., Angermeyer M.C., Corrigan P.W. (2005). Mental illness stigma: Concepts, consequences, and initiatives to reduce stigma. Eur. Psychiatry.

[104] Crowne D.P., Marlowe D. (1960). A new scale of social desirability independent of psychopathology. J. Consult. Psychol..

[105] Peris T.S., Teachman B.A., Nosek B.A. (2008). Implicit and explicit stigma of mental illness: Links to clinical care. J. Nerv. Ment. Dis..

[106] Dabby L., Tranulis C., Kirmayer L.J. (2015). Explicit and implicit attitudes of canadian psychiatrists toward people with mental illness. Can. J. Psychiatry.

[107] Teachman B.A., Wilson J.G., Komarovskaya I. (2006). Implicit and explicit stigma of mental illness in diagnosed and healthy samples. J. Soc. Clin. Psychol..

[108] Kopera M., Suszek H., Bonar E., Myszka M., Gmaj B., Ilgen M., Wojnar M. (2015). Evaluating Explicit and Implicit Stigma of Mental Illness in Mental Health Professionals and Medical Students. Commun. Ment. Health J..

[109] Cheon B.K., Chiao J.Y. (2012). Cultural Variation in Implicit Mental Illness Stigma Bobby. J. Cross. Cult. Psychol..

[110] Rudolph A., Hilbert A. (2015). A novel measure to assess self-discrimination in binge-eating disorder and obesity. Int. J. Obes..

[111] Dalky H.F. (2012). Mental Illness Stigma Reduction Interventions: Review of Intervention Trials. West. J. Nurs. Res..

[112] Thornicroft G., Mehta N., Clement S., Evans-Lacko S., Doherty M., Rose D., Koschorke M., Shidhaye R., O’Reilly C., Henderson C. (2016). Evidence for effective interventions to reduce mental-health-related stigma and discrimination. Lancet.

[113] Spagnolo A.B., Murphy A.A., Librera L.A. (2008). Reducing stigma by meeting and learning from people with mental illness. attitudes towards me. Psycnet. Apa. Org..

[114] Crisafulli M.A., Thompson-Brenner H., Franko D.L., Eddy K.T., Herzog D.B. (2010). Stigmatization of anorexia nervosa: Characteristics and response to intervention. J. Soc. Clin. Psychol..

[115] Crisafulli M.A., Von Holle A., Bulik C.M. (2008). Attitudes towards anorexia nervosa: The impact of framing on blame and stigma. Int. J. Eat. Disord..

[116] Iles I.A., Seate A.A., Waks L. (2016). Eating disorder public service announcements: Analyzing effects from an intergroup affect and stereotype perspective. Health Educ..

[117] Iles I.A., Atwell Seate A., Waks L. (2017). Stigmatizing the other: An exploratory study of unintended consequences of eating disorder public service announcements. J. Health Psychol..

[118] Christofi M., Michael-Grigoriou D. (2017). Virtual reality for inducing empathy and reducing prejudice towards stigmatized groups: A survey. Proceedings of the 2017 23rd International Conference on Virtual System & Multimedia (VSMM), Dublin, Ireland, 31 October–2 November 2017.

[119] Lucksted A., Drapalski A., Calmes C., Forbes C., DeForge B., Boyd J. (2011). Ending self-stigma: Pilot evaluation of a new intervention to reduce internalized stigma among people with mental illnesses. Psychiatr. Rehabil. J..

[120] Rüsch N., Xu Z. (2017). Strategies to Reduce Mental illness Stigma. The Stigma of Mental Illness-End of the Story.

[121] Strahan E.J., Stillar A., Files N., Nash P., Scarborough J., Connors L., Gusella J., Henderson K., Mayman S., Marchand P. (2017). Increasing parental self-efficacy with emotion-focused family therapy for eating disorders: A process model. Pers. Exp. Psychother..

